# Investigation of maternal and paternal perceptions of breastfeeding self-efficacy in the postpartum period in Turkey, 2023–2024: a cross-sectional study

**DOI:** 10.1590/1806-9282.20250778

**Published:** 2025-12-15

**Authors:** Hilal Kurt Sezer, Sibel Kucukoglu, Murat Konak

**Affiliations:** 1 Niğde Ömer Halisdemir University, Zübeyde Hanim Faculty of Health Sciences, Department of Nursing – Niğde, Turkey.; 2 Selçuk University, Faculty of Nursing – Konya, Turkey.; 3 Selçuk University, Faculty of Medicine – Konya, Turkey.

**Keywords:** Attitude, Breastfeeding, Maternal, Paternal

## Abstract

**OBJECTIVE:**

The aim of this study was to investigate maternal and paternal perceptions of breastfeeding self-efficacy in the postpartum period in Turkey between December 2023 and June 2024.

**METHODS:**

The present study was designed as a cross-sectional study. The sample consisted of 150 mothers and 150 fathers, making a total of 300 participants. Data were collected through face-to-face interviews using the "Descriptive Information Form for Mothers and Fathers," the "Breastfeeding Self-Efficacy Scale," and the "Paternal Breastfeeding Self-Efficacy Scale-Short Form." Data collection was carried out between December 2023 and June 2024. The obtained data were analyzed using the SPSS 25.0 statistical program, and a statistical significance level of p<0.05 was accepted.

**RESULTS:**

Mothers’ scores were higher than fathers’ scores (p<0.001). It was found that paternal breastfeeding self-efficacy significantly affected mothers’ breastfeeding self-efficacy (r=0.520, p<0.001). In the multiple regression analysis, monthly income, breastfeeding education, and the time of first breastfeeding were found to affect mothers’ breastfeeding self-efficacy (R^2^=0.099, p=0.02). Also found that employment status, breastfeeding experience, and planned pregnancy variables affected paternal breastfeeding self-efficacy (R^2^: 0.161, p<0.001).

**CONCLUSION:**

Empowering fathers about breastfeeding with in-service training and multidisciplinary approach is very important to increase breastfeeding rates. In addition, it is recommended that fathers should be handled together with pregnant women from the prenatal period, and counseling services should be provided to support the positive development of their perspective on breastfeeding.

## INTRODUCTION

Breast milk is the best food for infants, and the American Academy of Pediatrics (AAP) recommends exclusive breastfeeding for the first 6 months of life, followed by nutritious complementary foods and continued breastfeeding until 2 years of age and beyond^1^. Although it is recommended that infants should be exclusively breastfed for the first 6 months, it is noteworthy that breastfeeding rates are not at the desired level^2^. The Ministry of Health has been implementing the "Promoting Breastfeeding and Baby Friendly Hospitals" program since 1991^3^. However, there are still problems with exclusive breastfeeding. Breastfeeding is a process that both the infant and the parents need to adapt to. In addition to the mechanisms related to the infant in breastfeeding, the joint participation of mothers and fathers in the breastfeeding process also affects the duration and success of breastfeeding^4^. In addition, father's support during breastfeeding directly affects mothers’ breastfeeding self-efficacy^4,5^. Fathers need to assume many responsibilities to initiate breastfeeding in mothers and to maintain breastfeeding beyond 6 months. Lack of paternal support is a significant risk factor for early cessation of breastfeeding. This is an important issue that warrants emphasis and further investigation^4,6^. Therefore, this study aimed to investigate maternal and paternal perceptions of breastfeeding self-efficacy in the postpartum period in Turkey between December 2023 and June 2024. The study also examined in depth the factors that influence maternal and paternal breastfeeding self-efficacy and the extent to which they do so.

### Research questions

1. What are the breastfeeding self-efficacy levels of mothers?
2. What are the breastfeeding self-efficacy levels of fathers?
3. Is there a relationship between breastfeeding attitudes of mothers and fathers?
4. What are the variables affecting the breastfeeding attitudes of mothers and fathers?

## METHODS

### Study design

This cross-sectional study was conducted with 150 mothers and 150 fathers with actively breastfed infants living in a provincial center. The STROBE (Strengthening the Reporting of Observational Studies in Epidemiology) guideline was used to report the data of the study.

### Study setting and participants

The study was conducted in the outpatient clinics of child health and diseases in a Training and Research Hospital in a provincial center. The sample size for the study was determined by G. Power program. Since there were no similar studies using the same scales, an effect size of 0.25 (small), a margin of error of 0.05, and a power of 0.90 were selected from Cohen's standardized effect sizes. It was determined that it would be sufficient to include at least 130 participants in the groups. The study was completed with 150 mothers and 150 fathers. Mothers and fathers who (i) had a breastfed infant (0–6 months), (ii) volunteered, and (iii) had no communication problems were included in the study. Mothers who could not actively breastfeed and their spouses were excluded from the study.

### Measures

The dependent variables of the study were mothers’ and fathers’ breastfeeding self-efficacy scores, and the independent variables were sociodemographics.

### Data collection tools

The data of the study were collected with "Personal Information Form for Mothers and Fathers," "Postpartum Breastfeeding Self-Efficacy Scale," and "Paternal Breastfeeding Self-Efficacy Scale-Short Form."

Personal Information Form for Mothers and Fathers: This form, which was created by the researcher by reviewing the literature, was created separately for mothers (mother’ sociodemographic questions are: mother age, education level, employment status, monthly income, breastfeeding experience, first breastfeeding time, and the pregnancy’ state of being planned) and fathers (fathers’ sociodemographic questions are: father age, education level, employment status, monthly income, his partner's experience with breastfeeding, first breastfeeding time, and the pregnancy’ state of being planned)^4,5^.

Breastfeeding self-efficacy scale (BSES): The scale measures the extent to which mothers perceive themselves to be competent in breastfeeding^7^. All items in the scale have a positive meaning. The minimum score that can be obtained from the scale is 14, the maximum score is 70, and the scale has no cut-off point. The higher the scale score, the higher the breastfeeding self-efficacy. The Turkish adaptation of the scale was conducted by Aluş-Tokat and Okumuş in 2010, and the Cronbach alpha value was found to be 0.94^8^. In this study, Cronbach's alpha was 0.87.

Paternal Breastfeeding Self-Efficacy Scale-Short Form (PBSES-SH): The scale was developed by Dennis and Abbas Dick^9^. It has 14 items. There are no subdimensions in the scale, and scoring is done from 1 to 5. In the adaptation study of the scale done by Kucukoglu et al., the Cronbach alpha value was found to be 0.93^6^. In this study, the Cronbach alpha value was found to be 0.94.

### Ethical consideration

Ethics committee and institutional permission were obtained before the study. During the study, the participants were informed about the research, and their consent was obtained. This study was conducted in accordance with the principles of the Declaration of Helsinki.

### Statistical analysis

Data were analyzed using the IBM SPSS Statistics 25.0 software package. Normality distribution was examined using the Kolmogorov-Smirnov test. Student's t-test was used to compare the scale scores. The effects of sociodemographic variables and scales were tested by the multiple linear regression analysis. The results of the regression model were tabulated using B (95%CI), standard error (SE), estimated β, adjusted R^2^, F-test, and p-value for each variable. The Pearson correlation value was used as the basis for the relationship of the scales. Statistical significance was set at p<0.05.

## RESULTS

The demographic characteristics of the mothers and fathers are presented in Table 1. While the breastfeeding self-efficacy scores of the mothers participating in the study were 56.64±8.34, the scores of the fathers toward breastfeeding were 46.36±13.26. It was found that mothers’ breastfeeding self-efficacy scores were significantly higher than fathers’ (t=11.388, p<0.001) (Table 1).

**Table 1:** Comparison of demographic characteristics and scale scores [mothers (n=150) and fathers (n=150)].

Characteristics of mothers		N	%	Characteristics of fathers		n	%
Age		X±SD (29.00±5.79)		Age		X±SD (34.70±6.65)	
**Education**				**Education**			
	Primary education	49	30.6		Primary education	45	28.1
	High school	65	40.6		High school	55	34.4
	University and above	46	28.8		University and above	60	37.5
**Employment status**				**Employment status**			
	Yes	41	25.6		Yes	135	84.4
	No	119	74.4		No	25	15.6
**Monthly income**				**Monthly income**			
	Poor	100	62.5		Poor	45	28.1
	Middle	31	19.4		Middle	33	20.7
	Good	29	18.1		Good	82	51.2
**Breastfeeding experience (partner's)**		**N**		**%**			
	Yes	90		28.1			
	No	70		21.9			
**First breastfeeding time**							
	In 30 min	89		55.6			
	31–60 min	41		25.6			
	>61 min	22		13.8			
	Did not occur on the first day	8		5.0			
**The pregnancy's state of being planned**							
	Yes	121		75.6			
	No	39		24.4			
**Breastfeeding self-efficacy levels**			**X±SD**	**Mean difference**			**Test (p)**
	Mothers		56.64±8.34	10.28±11.42			**t=11.388 p<0.001** *
	Fathers		46.36±13.26				

t: independent samples t test;

* p<0.05;

X±SD: mean±standard deviation; %: percentage; n: number. Bold values indicate statistically significant results (p<0.05).

When the relationship between the breastfeeding scores of the mothers and fathers participating in the study was analyzed, it was seen that there was a statistically significant and positive moderate relationship between them (r=0.520, p<0.001) (Table 2).

**Table 2:** The relationship between the mean scale scores of mothers (n=150) and fathers (n=150).

	Breastfeeding self-efficacy (Mothers)	
	r (95%CI)	P
Breastfeeding self-efficacy (fathers)	0.520 (0.368; 0.654)	**p<0.001** *

r: correlation coefficient;

* p<0.05;

95%CI: confidence interval. Bold values indicate statistically significant correlations (p<0.05).

Multiple regression analysis (enter method) was performed to evaluate the effect of the variables determined to have an effect on mothers’ breastfeeding self-efficacy and paternal breastfeeding self-efficacy (Table 3). It was found that the variables belonging to mothers explained the change (variance) of breastfeeding self-efficacy score by 9% (R^2^). According to the results of the t-test for the significance of the regression coefficients, the order of significance of the three variables was found to be effective according to the standardized regression coefficient (Beta=b) as follows: monthly income, breastfeeding experiences, and breastfeeding initiation time (first breastfeeding time) (p<0.05). It was determined that the independent variables with positive values in the model, increasing monthly income, and having breastfeeding experience increased mothers’ breastfeeding self-efficacy scores (β=1.493, β=2.825). It was determined that the prolongation of the time of first initiation of breastfeeding with negative values (β=-1.612) affected the breastfeeding self-efficacy score of mothers in the direction of decreasing (Table 3).

**Table 3:** Examining the variables affecting the scale scores of mothers (n=150) and fathers (n=150) with multiple regression analysis.

Dependent variable	Independent variables	β	SE	Zβ	T	p	β %95CI		Model significance		
							Below	Above	F	p	R^2^
Breastfeeding self-efficacy (mothers)	Fixed	49.295	4.154		**11.866**	**0.000** *	41.087	57.503	2.391	**0.024** *	0.099
	Mother age	0.108	0.120	0.075	0.901	0.369	-0.129	0.344			
	Mother's education level	0.621	1.129	0.058	0.550	0.583	-1.609	2.851			
	Mother's employment status	0.829	1.757	0.048	0.472	0.638	-2.642	4.299			
	Monthly income	1.493	0.981	0.140	**2.122**	**0.030** *	0.446	3.432			
	Breastfeeding experience	2.825	1.379	0.169	**2.049**	**0.042** *	0.101	5.550			
	First breastfeeding time	-1.612	0.838	-0.153	**-2.024**	**0.046** *	-3.267	-0.043			
	The pregnancy’ state of being planned	1.530	1.544	0.079	0.991	0.323	-1.520	4.581			
Breastfeeding self-efficacy (fathers)	Fixed	29.230	5.514		**5.301**	**0.000** *	18,336	40.124	4.174	**<0.001** *	0.161
	Father age	0.814	1.873	0.045	0.435	0.664	-2.886	4.514			
	Father's education level	0.623	2.295	0.021	0.272	0.786	-3.911	5.158			
	Father's employment status	5.364	3.607	0.118	**2.187**	**0.039** *	1.763	12.491			
	Monthly income	1.713	1.627	0.101	1.053	0.294	-1.502	4.929			
	Breastfeeding experience (partner's)	4.248	2.012	0.159	**2.111**	**0.036** *	0.272	8.224			
	First breastfeeding time	-1.313	1.283	-0.079	-1.023	0.308	-3.848	1.222			
	The pregnancy’ state of being planned	8.849	2.487	0.287	**3.557**	**0.000** *	3.935	13.764			

Dependent variable: Breastfeeding self-efficacy scale for mothers, Breastfeeding self-efficacy scale for fathers short form scores;

* p<0.05; CI: confidence interval; SE: standard error; β: regression coefficient; zβ: standardized regression coefficient; β 95%CI: 95% confidence interval for the regression coefficient; F: regression test value; R^2^: determination coefficient; T: significance of the regression coefficient; Below: below value; Above: above value. Bold values indicate statistically significant predictors (p<0.05).

According to the multiple regression analysis, the demographic variables found to be significant for fathers explained 16% of the variation (variance) in breastfeeding attitudes. According to the results of the t-test for the significance of the regression coefficients, the order of significance of the three variables found to be effective according to the standardized regression coefficient (Beta=b) is as follows: Fathers’ employment status, breastfeeding experience (p<0.05), and the pregnancy’ state of being planned (p<0.001, more effective). Whether the planned pregnancy, fathers’ employment status, and breastfeeding experience tended to increase paternal breastfeeding self-efficacy (Table 3).

## DISCUSSION

In the postnatal period, it is necessary to determine the support of fathers as well as mothers in breastfeeding. Although the benefits of fathers’ participation in the breastfeeding process are emphasized by many studies, it is noteworthy that the active participation of fathers in the breastfeeding process is not at the desired level^6^. In this study, which was planned to determine fathers’ competencies in supporting their wives during breastfeeding and to compare these results with mothers’ breastfeeding self-efficacy perceptions, it was found that mothers’ breastfeeding self-efficacy was better than fathers’ attitude toward breastfeeding.

It was determined that mothers’ breastfeeding self-efficacy levels were higher than paternal breastfeeding self-efficacy levels (p<0.001). In some studies, in the literature, it is pointed out that although fathers want to support their wives during breastfeeding, they cannot trust themselves. Similar to our study, these findings support the finding that paternal breastfeeding self-efficacies were lower than those of mothers^10^. In a different study, it was also found that spousal support had no effect on the duration of breastfeeding in breastfeeding mothers^11^. For fathers, breastfeeding self-efficacy refers to their perception of their ability to help their partner in the breastfeeding process and their self-confidence. Although there are different study findings, it is very important to include fathers in interventions to increase breastfeeding rates of mothers and breastfeeding self-efficacy^9^.

When the relationship between the breastfeeding scores of the mothers and fathers participating in the study was examined, it was determined that there was a statistically significant relationship between them (p<0.001). Fathers’ positive attitudes toward breastfeeding increase mothers’ self-efficacy. Similarly, Klaravina and Wulan emphasized that partner support is an important factor in improving breastfeeding^12^. In Davidson and Ollerton's integrated review study, it was pointed out that spousal support supported autonomy and self-efficacy in women^13^. In the systematic review published by Koksal et al., it was determined that involving fathers in the breastfeeding process and ensuring their participation could increase breastfeeding rates^14^. In another published study, it was stated that fathers should be encouraged to actively participate in breastfeeding and be informed about breastfeeding^15^. In some studies, stated that increased income, employment, breastfeeding history, and planned pregnancy were reported to be variables that may affect breastfeeding like this study^6,14,16,17^. It is seen that spousal support has an effect on breastfeeding success in order to positively affect mothers’ breastfeeding self-efficacy. It should be kept in mind that high breastfeeding self-efficacy is a factor that may affect the rate of exclusive breastfeeding for the first 6 months^18,19^.

### Limitations

The strengths of this study are its use of a quantitative design that examines fathers’ and mothers’ breastfeeding self-efficacy and the relationship between these variables using multiple regression analysis. Another strength of the study is the inclusion of fathers and mothers during the first 6 months of life, when breastfeeding frequency is high. One of the limitations of the study is that it was registered in a single center. Another limitation is that although the self-efficacy of mothers and fathers regarding breastfeeding was assessed, the feeding of the infants could not be monitored.

## CONCLUSION

Although breastfeeding has many benefits on child health, breastfeeding rates are not at the desired level. In order to improve breastfeeding rates, mothers and fathers should be empowered together from the prenatal period. Considering the effect of fathers on breastfeeding, it is very important for mothers to feel competent in breastfeeding in order to continue breastfeeding for the first 6 months. It is seen that there is a need for studies with different designs and focus group interviews in which breastfeeding self-efficacy and attitudes of mothers and fathers are examined in depth.

## Data Availability

The datasets generated and/or analyzed during the current study are available from the corresponding author upon reasonable request.
